# Supplementary material for: The transcriptomic landscape of spinal V1 interneurons reveals a role for En1 in specific elements of motor output
Source: bioRxiv. 2024 Oct 26:2024.09.18.613279. Originally published 2024 Sep 19. Preprint. [Version 2] doi: 10.1101/2024.09.18.613279 (PMC11429899; doi:10.1101/2024.09.18.613279)
Supplement: Supplement 1 [file NIHPP2024.09.18.613279v2-supplement-1.pdf]

bioRxiv preprint doi: https://doi.org/10.1101/2024.09.18.613279; this version posted October 26, 2024. The copyright holder for this preprint (which was not certified by peer review) is the author/funder, who has granted bioRxiv a license to display the preprint in perpetuity. It is made available under aCC-BY 4.0 International license.

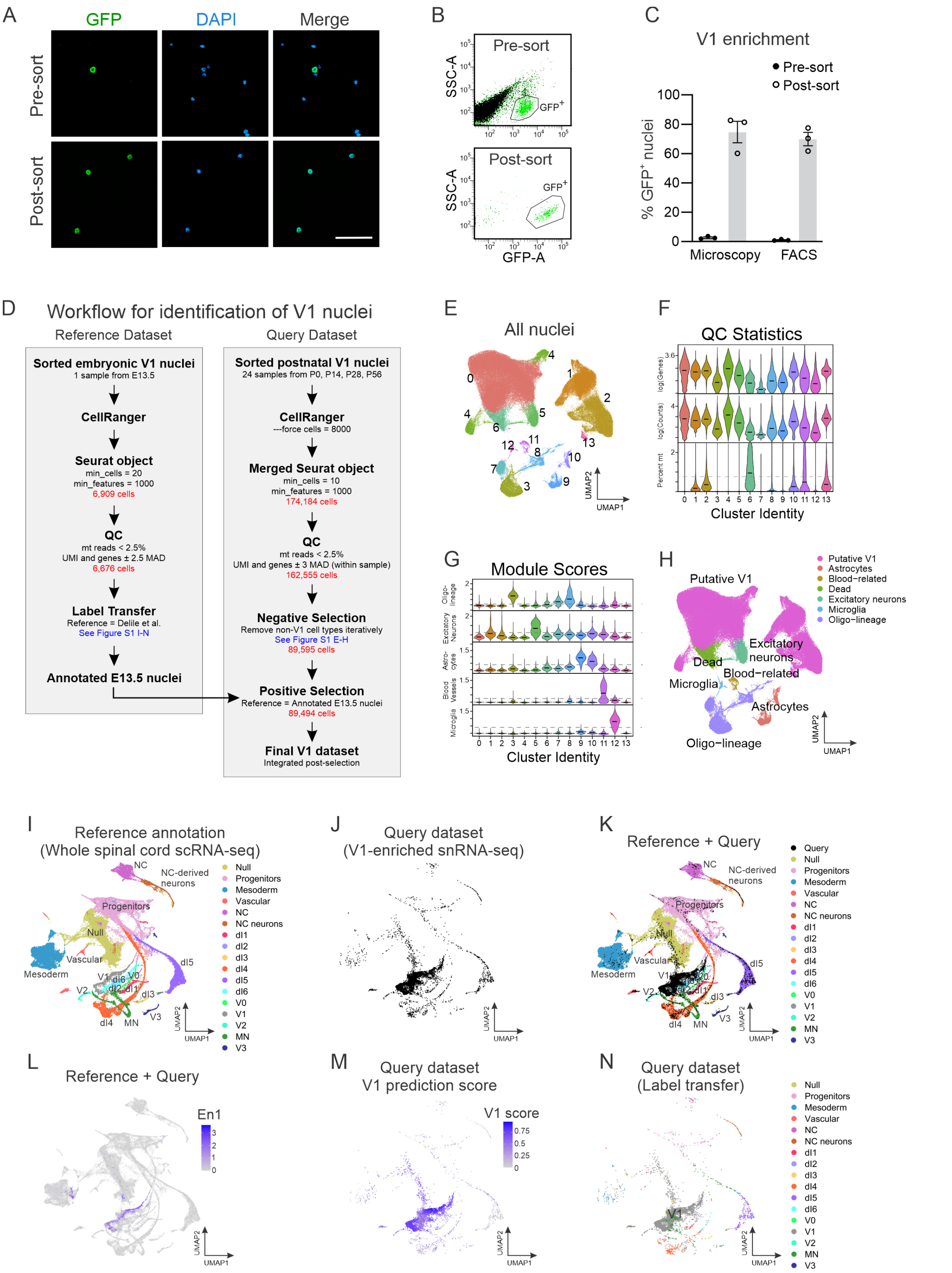

Figure S1. Enrichment of V1 nuclei via fluorescence activated cell sorting, and subsequent identification of V1 nuclei, related to Figure 1.

bioRxiv preprint doi: https://doi.org/10.1101/2024.09.18.613279; this version posted October 26, 2024. The copyright holder for this preprint (which was not certified by peer review) is the author/funder, who has granted bioRxiv a license to display the preprint in perpetuity. It is made available under aCC-BY 4.0 International license.

(A-C) Enrichment of V1 nuclei by fluorescence activated cell sorting (FACS). (A) Example images of nuclei isolated from P0 *En1::Cre; RC::lsl.Sun1-sfGFP* (*En1^{INTACT}*) spinal cords before (top) and after (bottom) FACS. Scale bar = 50 μm. B) Representative FACS analysis of spinal cord nuclei before sorting (top) and then re-analyzed after sorting (bottom). (C) Quantification of V1 nuclei enrichment assessed via microscopy or FACS before sorting (2.7% ± 0.5% and 1.1% ± 0.3%, respectively) and after sorting (74.7% ± 7.3% and 69.9% ± 4.6%, respectively) (n = 3 independent trials, mean ± SEM).
(D) Workflow outlining the identification of V1 nuclei within the postnatal nuclei dataset (Query Dataset) by using both negative and positive selection criteria.
(E-H) Negative selection to remove contaminating (non-V1 interneuron) nuclei. (E) UMAP of all nuclei meeting quality control (QC) cutoffs across all ages, replicates, and anatomical regions combined. Colors indicate unbiased Louvain graph-based clustering. (F) The number of genes detected per nuclei, total reads per nuclei, and percent of mitochondrial reads are shown per cluster. Cluster 6 was identified as "Dead" due to the low number of genes and counts, and high number of mitochondrial reads. (G) Gene module scores per cluster for non-V1 cell types. Clusters above the threshold (dotted line) were identified as the corresponding cell type. See Supplementary Table 1 for genes used to create the module score. (H) The UMAP shown in (E) with clusters assigned an identity per the selection measures described in (F) and (G).
(I-N) Positive identification of V1 interneurons using label transfer. (I) E9.5-E13.5 whole spinal cord scRNA-seq data[22] was used as a reference data set. (J) E13.5 *En1::Cre^{INTACT}* snRNA-seq query data (black) enriched for V1 interneurons by flow cytometry. (K) Data set from (J) projected onto reference data (I). (L) Expression of the transcription factor En1 overlaid on the projected data set. (M-N) V1 label transfer prediction scores and final assignments for the E13.5 snRNA-seq data set, used for subsequent positive and negative selection of our postnatal data.

bioRxiv preprint doi: https://doi.org/10.1101/2024.09.18.613279; this version posted October 26, 2024. The copyright holder for this preprint (which was not certified by peer review) is the author/funder, who has granted bioRxiv a license to display the preprint in perpetuity. It is made available under aCC-BY 4.0 International license.

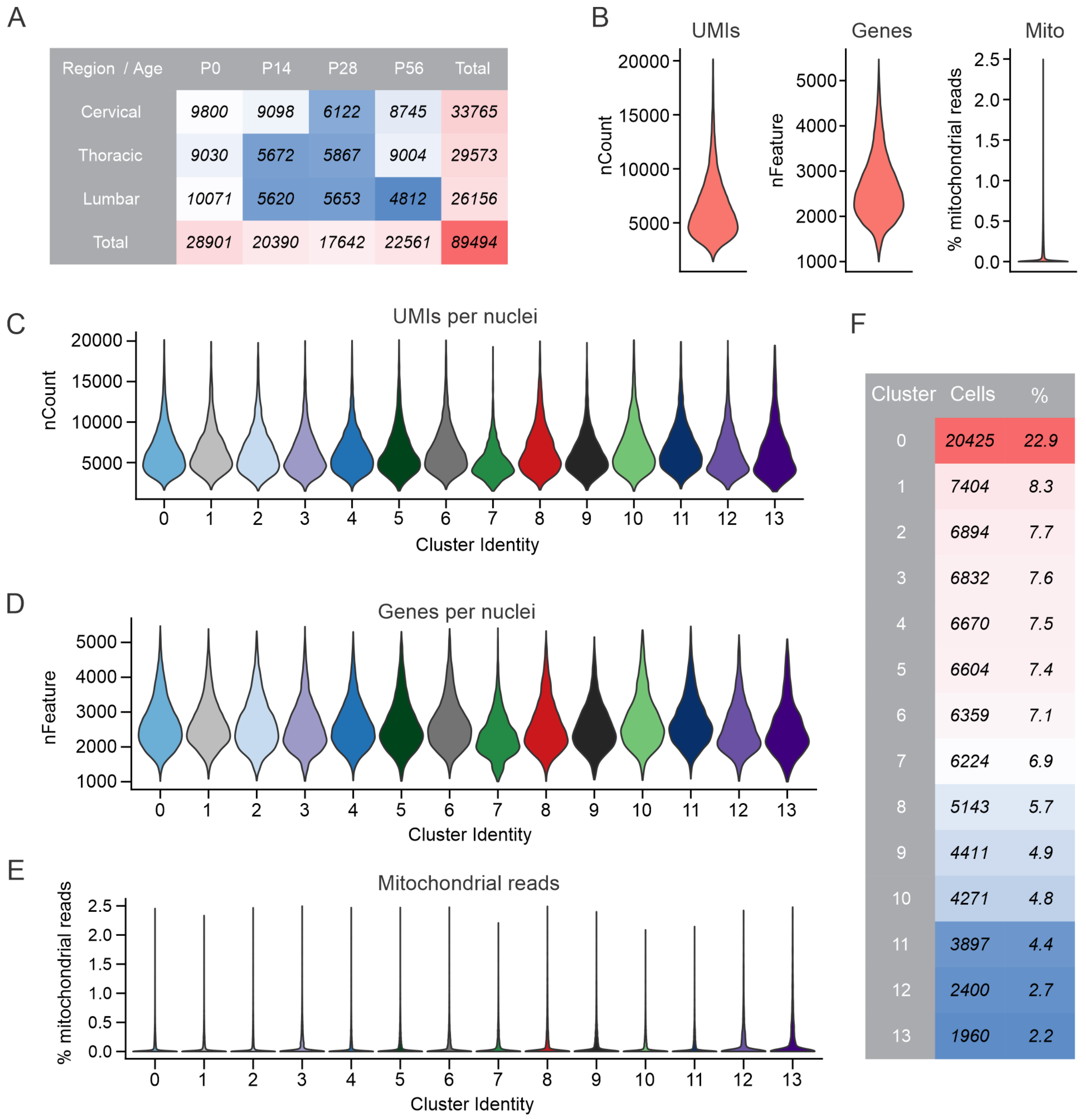

Figure S2. Quality Control (QC) statistics on V1 interneuron nuclei, related to Figure 1. (A) Table showing the total number of nuclei identified as V1 interneurons grouped by age and anatomical location. Colors represent low (blue) to high (red) proportions across all categories. (B) Overall QC statistics on V1 interneurons showing total number of unique molecular identifier (UMI)-corrected counts per nuclei, number of genes detected per nuclei, and the percentage of mitochondrial reads per nuclei. (C-E) Similar to (B) except the same statistics were parsed by the cluster identification shown in Figure 1C. (F) Table displaying the number and percentage of nuclei in each cluster.

bioRxiv preprint doi: https://doi.org/10.1101/2024.09.18.613279; this version posted October 26, 2024. The copyright holder for this preprint (which was not certified by peer review) is the author/funder, who has granted bioRxiv a license to display the preprint in perpetuity. It is made available under aCC-BY 4.0 International license.

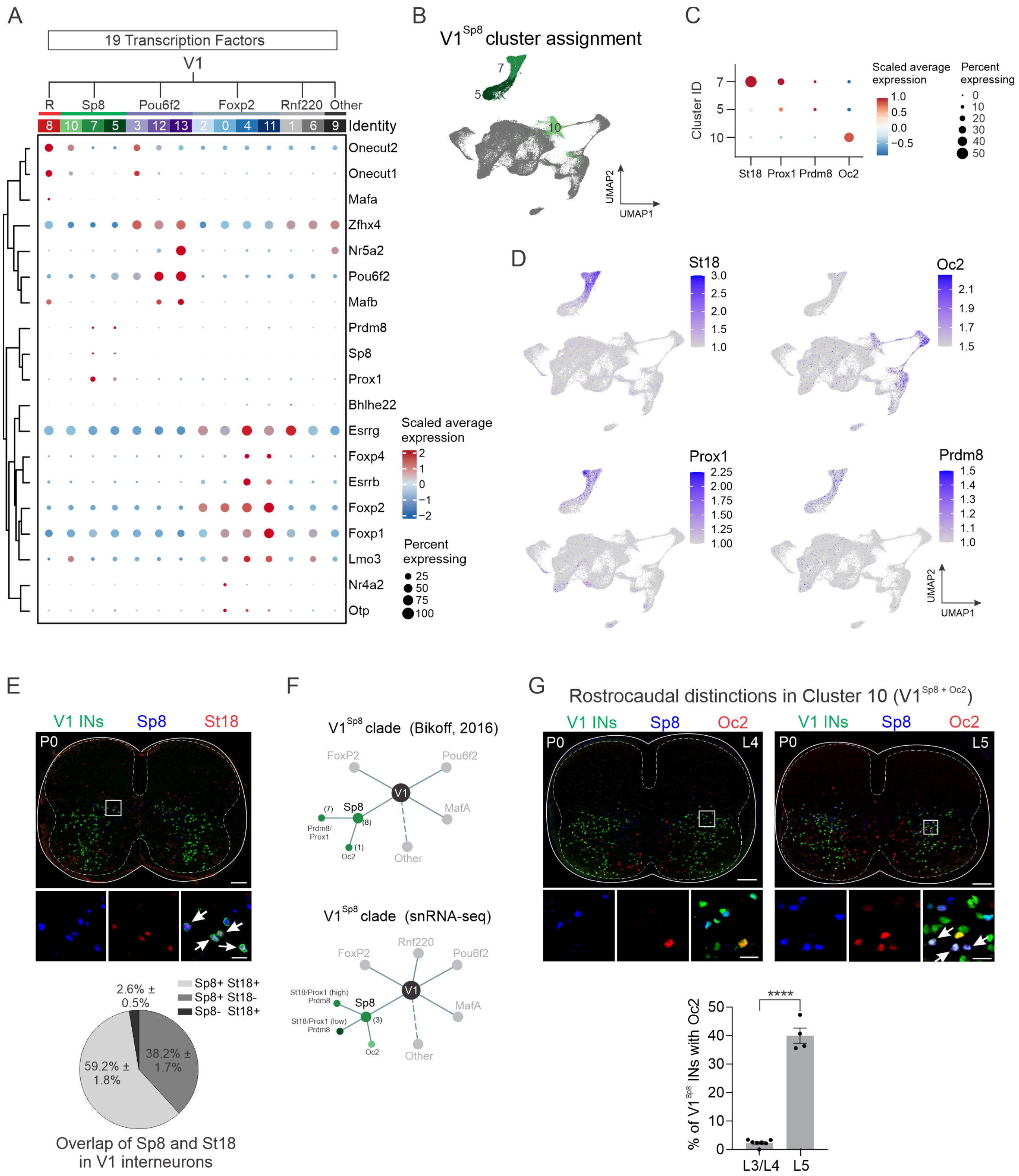

Figure S3. Analysis of transcription factor expression and V1^{Sp8} clade structure, related to Figures 1 and 3.

(A) Dot plot showing scaled average expression of the 19 transcription factors (TFs) described previously in Bikoff et al.,[30] all of which were validated via immunohistochemistry (IHC). Note that several genes were detected at low levels but still showed biased expression. (B) V1^{Sp8} cluster

bioRxiv preprint doi: https://doi.org/10.1101/2024.09.18.613279; this version posted October 26, 2024. The copyright holder for this preprint (which was not certified by peer review) is the author/funder, who has granted bioRxiv a license to display the preprint in perpetuity. It is made available under aCC-BY 4.0 International license.

assignment, as described in Figure 1F. (C) Corresponding dot plot highlighting St18, Prox1, Prdm8, and Oc2 expression within the $V1^{Sp8}$ clade. (D) UMAP plots of St18, Prox1, Prdm8, and Oc2 showing expression in V1 interneurons. (E) Top, IHC validation of St18 expression in V1 interneurons (arrows) in P0 lumbar spinal cord of *En1::Cre; RC.lsl.Sun1-sfGFP* mice. Scale bars = 100 μm (top) or 20 μm (inset). Bottom, the proportion of Sp8 and St18 co-expression in V1 interneurons as assessed by IHC. (F) Top, simplified $V1^{Sp8}$ clade diagram adapted from Bikoff, et al.[30] showing delineation of $V1^{Sp8}$ neurons into seven Prdm8/Prox1 subsets and a single Oc2-expressing subset. Bottom, revised clade diagram based on snRNA-seq data, showing general alignment with previous analyses. (G) Top, IHC analysis of cluster #10 in P0 lumbar spinal cord of *En1::Cre; Tau.lsl.nLacZ* mice. Scale bars = 100 μm (top) or 20 μm (inset). Bottom, $V1^{Sp8+Oc2}$ interneurons (arrows) were significantly enriched in caudal (L5) spinal segments compared to more rostral (L3/L4) segments, thus highlighting segmental differences in V1 cell types. ****p < 0.0001, unpaired t-test.

bioRxiv preprint doi: https://doi.org/10.1101/2024.09.18.613279; this version posted October 26, 2024. The copyright holder for this preprint (which was not certified by peer review) is the author/funder, who has granted bioRxiv a license to display the preprint in perpetuity. It is made available under aCC-BY 4.0 International license.

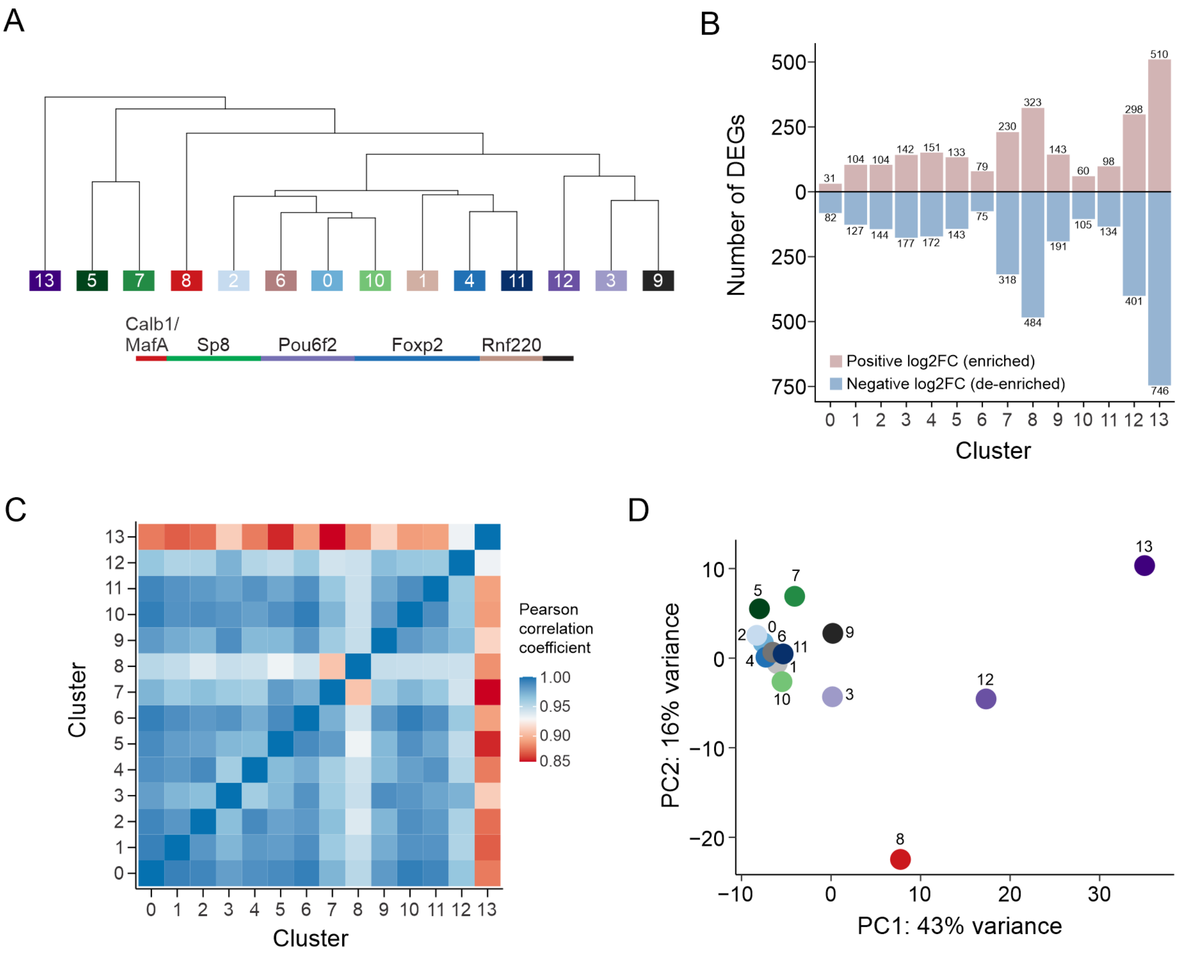

Figure S4. Taxonomic relationship and similarity of V1 clusters, related to Figure 3.
(A) Dendrogram showing phylogenetic analysis based on the top 3000 most variable genes. (B) Number of differentially expressed genes (DEGs) per cluster based on non-parametric Wilcoxon rank sum test for differential gene expression. (C) Heatmap of Pearson correlation coefficients based on the log-normalized average expression of all genes per cluster identified cluster #13 as unique. (D) Principal component analysis (PCA) plot of aggregated counts per cluster showing that cluster #13 drives most of the variance along PC1.

bioRxiv preprint doi: https://doi.org/10.1101/2024.09.18.613279; this version posted October 26, 2024. The copyright holder for this preprint (which was not certified by peer review) is the author/funder, who has granted bioRxiv a license to display the preprint in perpetuity. It is made available under aCC-BY 4.0 International license.

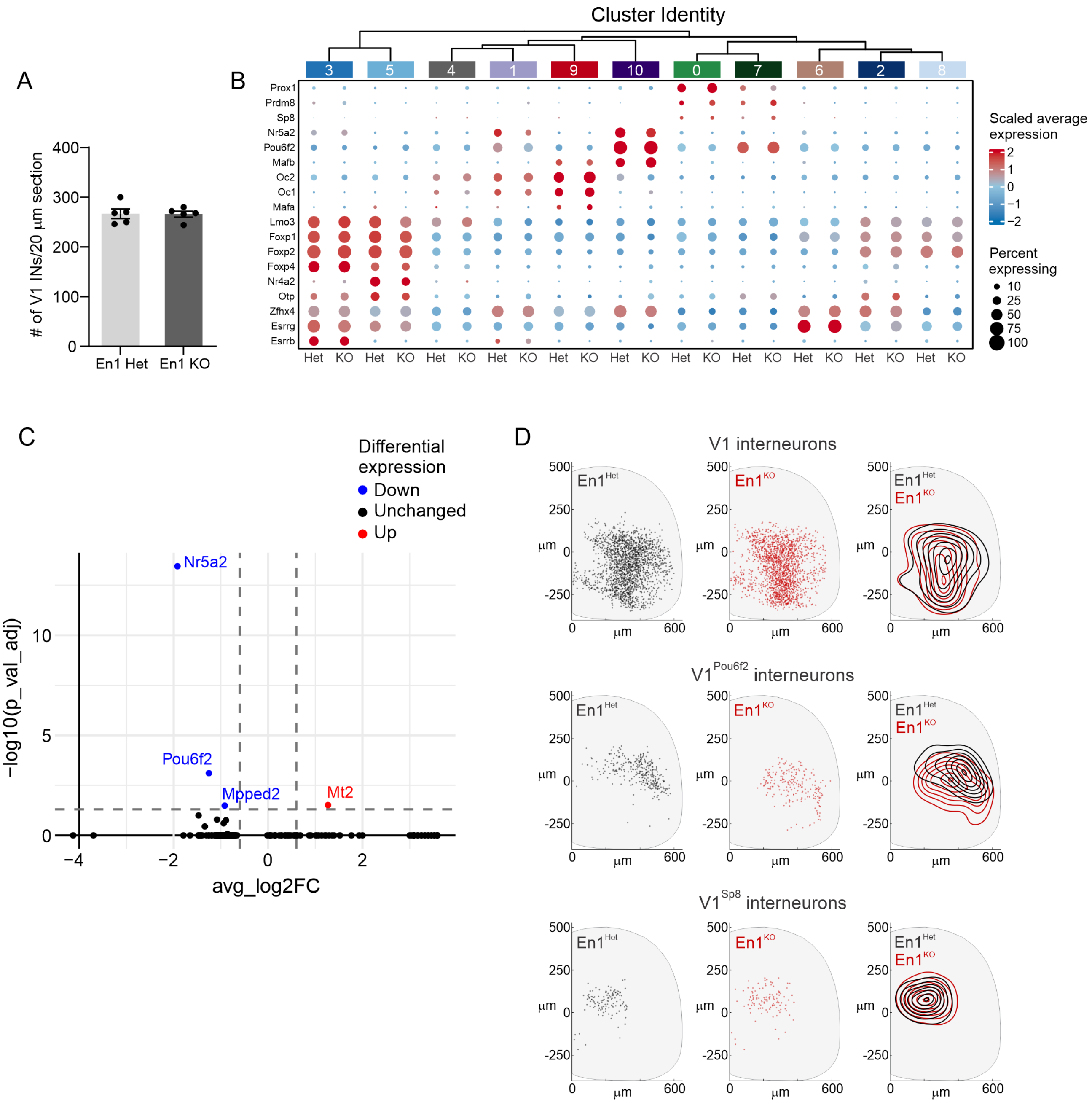

Figure S5. Additional gene expression analysis in *En1^{Het}* and *En1^{KO}* V1 interneurons, related to Figure 5.

(A) The total number of V1 interneurons is similar in *En1^{Het}* and *En1^{KO}* mice (p = 0.815, unpaired two-tailed t-test). (B) Dot plot showing similar gene expression of 19 transcription factors in V1 nuclei from *En1^{Het}* and *En1^{KO}* mice. Note that the remaining *En1^{KO}* V1 nuclei in cluster #10 retained a normal *Pou6f2^{+}* and *Nr5a2^{+}* identity. (C) Pseudo-bulk analysis of differentially expressed genes revealed that *Nr5a2* and *Pou6f2* are significantly downregulated in *En1^{KO}* V1 nuclei, compared with that in *En1^{Het}* nuclei. (D) Spatial distributions of the overall V1 interneuron population (top), V1^{Pou6f2} interneurons (middle), and V1^{Sp8} interneurons (bottom) in P0 lumbar

bioRxiv preprint doi: https://doi.org/10.1101/2024.09.18.613279; this version posted October 26, 2024. The copyright holder for this preprint (which was not certified by peer review) is the author/funder, who has granted bioRxiv a license to display the preprint in perpetuity. It is made available under aCC-BY 4.0 International license.

spinal segments of *En1*^{Het} (black) and *En1*^{KO} (red) mice. The position of V1^{Pou6f2} neurons was altered in *En1*^{KO} mice, whereas the position of V1^{Sp8} interneurons was unaffected (2-dimensional Kolmogorov-Smirnov test: $p = 6.5 \times 10^{-13}$ for V1^{Pou6f2} neurons; $p = 0.095$ for V1^{Sp8} neurons). Distributions were based on the following sample size: *En1*^{Het} (V1): n = 4 animals, 13 hemisections, 1776 neurons from L3-L5 segments; *En1*^{KO} (V1): n = 5 animals, 13 hemisections, 1702 neurons from L3-L5 segments; *En1*^{Het} (V1^{Pou6f2}): n = 8 animals, 21 hemisections, 309 neurons from L3 segments; *En1*^{KO} (V1^{Pou6f2}): n = 5 animals, 21 hemisections, 213 neurons from L3 segments; *En1*^{Het} (V1^{Sp8}): n = 3 animals, 8 hemisections, 122 neurons from L3-L5 segments; and *En1*^{KO} (V1^{Sp8}): n = 3 animals, 6 hemisections, 113 neurons from L3-L5 segments.

bioRxiv preprint doi: https://doi.org/10.1101/2024.09.18.613279; this version posted October 26, 2024. The copyright holder for this preprint (which was not certified by peer review) is the author/funder, who has granted bioRxiv a license to display the preprint in perpetuity. It is made available under aCC-BY 4.0 International license.

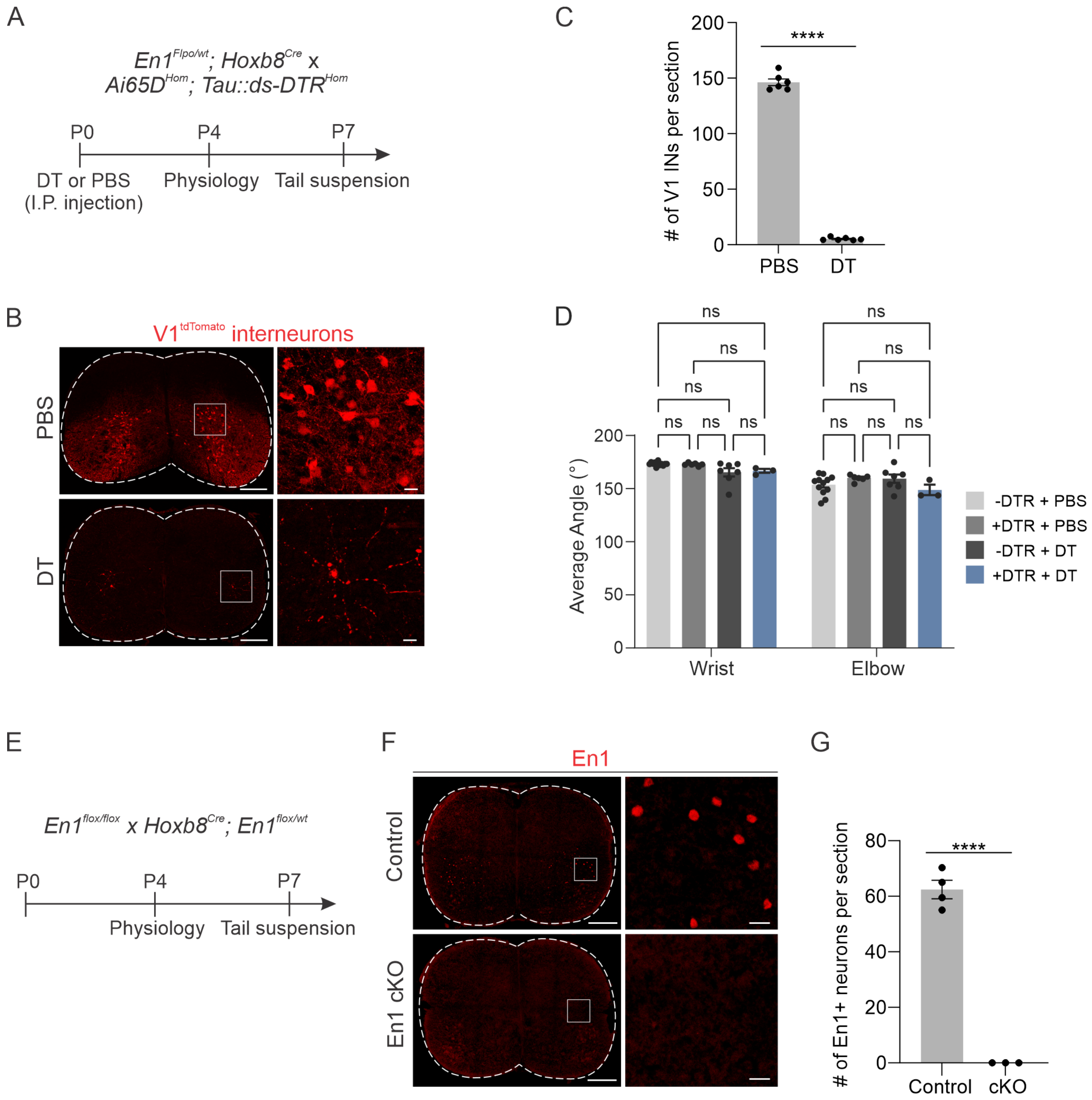

Figure S6. Validation of V1 ablation and *En1* conditional knockout experiments, and additional behavioral data, related to Figure 6.

(A) Schematic of the V1 ablation paradigm. Diptheria toxin (DT) was administered at P0, followed by either physiological analysis via fictive locomotion 4 days post-DT administration, or limb kinematic analysis during tail suspension 7 days post-DT administration. All mice in the study were heterozygous for the *Tau::ds-DTR* and *Ai65D* alleles. Littermates lacking Cre, Flpo, or both were pooled and used as controls. (B-C) Immunohistochemical analysis and quantification in P7 lumbar spinal cords of control (+PBS) or V1-ablated (+DT) quadruple-heterozygous mice showed near complete ablation of V1 interneurons at P7 (n = 6 mice, ****p < 0.0001, unpaired two-tailed

bioRxiv preprint doi: https://doi.org/10.1101/2024.09.18.613279; this version posted October 26, 2024. The copyright holder for this preprint (which was not certified by peer review) is the author/funder, who has granted bioRxiv a license to display the preprint in perpetuity. It is made available under aCC-BY 4.0 International license.

t-test). Scale bars = 200 μm or 20 μm (inset) (D) The average angles of the wrist and elbow joints were not significantly changed upon ablation of V1 interneurons (n = 3-12 mice, mean ± SEM, two-way ANOVA followed by Tukey HSD test, ns = not significant). (E) Schematic of the experimental paradigm for physiological and behavioral analyses of *En1* conditional knockout (*En1 cKO*) mice. (F-G) Immunohistochemical analysis (F) and quantification (G) showing complete loss of En1 expression in *En1 cKO* (*Hoxb8::Cre; En1*$^{flox/flox}$) mice in P0 lumbar spinal cord (n = 3-4 mice, *****p* < 0.0001, unpaired two-tailed *t*-test). Scale bars = 200 μm or 20 μm (inset).

TABLES

Table S1. Quality control metrics for CellRanger, related to Figures 1- 5.

Table S2. Cell-type classification markers, related to Figures 1-5.

Table S3. Cell barcodes of analyzed V1 nuclei, related to Figures 1-5.

Table S4. Differentially expressed genes in V1 clusters, related to Figures 1-3.

Table S5. Differentially expressed genes by developmental age, related to Figure 4.

Table S6. Differentially expressed genes in V1 clusters in the *En1*^{Het} versus *En1*^{KO} experiment, related to Figure 5.

VIDEOS

Video S1. Examples of tail suspension assay in control, V1-ablated, and *En1 cKO* mice, related to Figure 6.
